# Prognostic significance of PIK3CA and SOX2 in Asian patients with lung squamous cell carcinoma

**DOI:** 10.3892/ijo.2014.2742

**Published:** 2014-11-10

**Authors:** YOSHIHITO IIJIMA, MASAHIRO SEIKE, RINTARO NORO, TAKAYUKI IBI, SHINGO TAKEUCHI, IWAO MIKAMI, KIYOSHI KOIZUMI, JITSUO USUDA, AKIHIKO GEMMA

**Affiliations:** 1 Department of Thoracic Surgery, Graduate School of Medicine, Nippon Medical School, Tokyo 113-8603, Japan; 2 Department of Pulmonary Medicine and Oncology, Graduate School of Medicine, Nippon Medical School, Tokyo 113-8603, Japan; 3 Department of Thoracic Surgery, Aizu Chuo Hospital, Fukushima 965-8611, Japan

**Keywords:** PIK3CA, SOX2, lung squamous cell carcinoma, prognosis

## Abstract

The recent development of human genome studies has demonstrated the possibility of alteration of several genes as oncogenic driver mutations of lung squamous cell carcinoma (SQCC). FGFR1, PIK3CA and SOX2 genes have been recognized as candidate driver genes of SQCC. The aim of the present study was to evaluate FGFR1, PIK3CA and SOX2 protein expression in SQCC and determine whether the expression of these can be used as prognostic biomarkers. We evaluated the relationships between FGFR1, PIK3CA and SOX2 expression by immunohistochemical analysis and overall survival in lung SQCC patients with stage I–III that originated from China, United States and Japan. FGFR1-positive, PIK3CA-negative and SOX2-positive staining each showed trends toward better survival, although the differences were not statistically significant in a Chinese cohort of 57 patients. Patients with PIK3CA-negative and SOX2-positive staining (PIK3CA^−^/SOX2^+^) showed better prognosis compared with those with PIK3CA-positive or SOX2-negative staining in the Chinese cohort (P=0.04). The robustness of PIK3CA^−^/SOX2^+^ classification as having prognostic significance was validated in an independent set of 66 Japanese cohort patients (P=0.007). Japanese SQCC patients with stage I were evaluated separately and PIK3CA^−^/SOX2^+^ cases had significantly better survival than the group with PIK3CA-positive or SOX2-negative status (P=0.03). In univariate and multivariable Cox proportional hazards models of Asian stage I patients, the PIK3CA^−^/SOX2^+^ classification was statistically significantly associated with survival and was an independent prognostic factor. Classification by PIK3CA and SOX2 protein expression is useful for predicting the prognosis of Asian patients with lung SQCC with stage I.

## Introduction

Lung cancer is the leading cause of cancer death in Japan and worldwide (1). The majority of lung cancers are non-small cell lung cancers (NSCLC) that include adenocarcinomas (ADC) and squamous cell carcinomas (SQCC). Recently, oncogenic driver mutations including EGFR gene mutation and ALK fusion gene have been found in the majority of ADC (2–4). Recent randomized trials of gefitinib, erlotinib and crizotinib have demonstrated the significant superiority of these molecular targeted drugs on progression-free survival compared with standard chemotherapies as the key agents for the treatment of advanced ADC with driver mutations (5–7). However, oncogenic driver mutations are not typically present in SQCC. Molecular targeted agents developed for lung ADC are largely ineffective against lung SQCC. The standard of care for advanced SQCC patients is still cytotoxic chemotherapy. Unfortunately, modern cytotoxic and molecular targeted agents, pemetrexed and bevacizumab, are not recommended for patients with SQCC (8,9). Therefore, identification of biomarkers predictive of drug sensitivity and personalized therapies using the biomarkers could have a clinically significant impact on treatment strategies for SQCC.

Surgical resection is the standard treatment for early-stage NSCLC. Adjuvant chemotherapy after surgery is recognized as a standard therapy for resected stage IB, II and IIIA NSCLC. Three large randomized clinical trials have demonstrated a survival benefit for adjuvant cisplatin (CDDP)-based chemotherapy compared with surgery alone in patients who underwent surgery for NSCLC (10–12). While adjuvant chemotherapy has improved survival for patients with early-stage NSCLC, the prognosis of NSCLC after recurrence remains poor, in particular SQCC. Numerous promising biomarkers are currently being evaluated in the adjuvant setting. Among the molecular markers used as potential predictors of a survival benefit from adjuvant chemotherapy, ERCC1 and RRM1 have been reported (13,14). However, it is controversial whether ERCC1 predicts the prognosis of lung cancer treated with CDDP-based chemotherapy (15). Molecular biomarkers for adjuvant therapy decision should be identified in NSCLC patients, in particular SQCC patients. Patient selection based on the presence of prognostic or predictive biomarkers offers the potential to improve survival in SQCC patients.

Recent human genome studies have demonstrated the possibility of alteration of several genes as oncogenic driver mutations of SQCC (16–22). Multiplex testing for driver mutations in SQCC of the lung (SQ-MAP) using specimens from 40 SQCC patients was reported in 2012 (17). FGFR1 amplification was found in 25% and PIK3CA mutation was observed in 11% of SQCC tumors by SQ-MAP. Based on the SQ-MAP, two approved clinical trials using FGFR1 inhibition and PI3K inhibition have begun. In addition, the Cancer Genome Atlas Research reported that overexpression and amplification of SOX2 were observed in 21% and PIK3CA gene alteration was found in 16% of 178 lung SQCC samples (18). Chromosome 3q26 is frequently amplified in lung SQCC, showing the most frequent overexpression of two known oncogenes on 3q26: SOX2 and PIK3CA (20,21). Based on these findings, FGFR1, PIK3CA and SOX2 have been recognized as candidate driver genes of SQCC. However, the association between these genes including FGFR1, PIK3CA and SOX2 and patients’ prognosis has not been clarified.

In the present study, we evaluated whether three proteins, FGFR1, PIK3CA and SOX2, could be used as prognostic factors in SQCC patients from three country cohorts by immunohistochemical analysis (IHC). We ultimately found that the combination of PIK3CA and SOX2 protein expression could be useful for the prognosis of Asian patients with SQCC, in particular stage I patients. Our prediction criteria may be applicable to selection of SQCC patients who would benefit from adjuvant chemotherapy.

## Materials and methods

### Tissue microarray and clinical samples

Commercially available paraffin-embedded sections of the tissue microarray (TMA) of Chinese patients (OD-CT-RsLug01-009; Shanghai Outdo Biotech Co., Ltd., Shanghai, China) was used as the training set (Table I). Fifty-seven SQCC specimens were available in this array. Another TMA from American TA116 (TriStar, Rockville, MD, USA) was used as the validation set. Fifty-two SQCC specimens were available in this array.

We used a validation set of lung tumor tissue samples from 66 Japanese patients with stage I–III SQCC who had undergone surgical resection at the Nippon Medical School Hospital from 2001 to 2008. All tissues were freshly collected during surgery, snap-frozen and stored at −80°C. TNM stage, T factor, N factor, Grade, Ly factor and V factor were classified according to the World Health Organization TNM staging 7th edition. Information on patient survival and recurrence during 5 years of follow-up was available for all 66 cases (Table II). Twenty-eight patients received adjuvant chemotherapy with uracil-tegafur (UFT). The lung tissues from lung SQCC patients were used only for immunohistochemical analysis. Immunohistochemical staining of the lung cancer tissue samples was carried out in accordance with the principles embodied in the Declaration of Helsinki, 2008. All included patients provided written informed consent for the use of their tissue specimens for medical research.

### Immunohistochemistry

Immunohistochemical staining was performed on paraffin-embedded sections. After deparaffinization, antigen retrieval was carried out in 10 mmol/l citrate buffer (pH 6.0) (LSI Medience Corp., Tokyo, Japan) using an autoclave. After blocking swine serum albumin (1:50 dilution; Vector Laboratories Inc., Burlingame, CA, USA), the sections were washed and incubated with rabbit anti-human FGFR1 polyclonal antibody (1:250 dilution; Abcam Biochemicals, Cambridge, MA, USA) or with rabbit anti-human PIK3CA polyclonal antibody (1:50 dilution; Santa Cruz Biotechnology, Dallas, TX, USA) or with rabbit anti-human SOX2 polyclonal antibody (1:500 dilution; Millipore, Bedford, MA, USA) at 4°C overnight. After washing, they were incubated with biotinylated goat anti-rabbit IgG (1:200 dilution; Vector Laboratories) for 30 min. Finally, they were incubated with avidin-biotin complex kit (Funakoshi, Tokyo, Japan). Negative controls were prepared by omitting the primary antibody under the same experimental conditions.

### Evaluation of FGFR1, SOX2 and PIK3CA protein expression

Immunohistochemical scoring was performed using the Histoscore (H-score) (23). FGFR1 expression level was scored on a scale as follows: no expression (0), low expression according to a previous study (1+) and high expression (2+ and 3+). PIK3CA staining was scored on a scale as follows according to a previous study (24): 0, no staining; 1+, staining <50%; 2+, ≥50% with weak intensity: 3+, ≥50% with strong intensity. Nuclear expression of SOX2 protein was scored semiquantitatively by the combination of intensity (scored as 0, no staining; 1, weak staining; 2, moderate staining; 3, strong staining) and proportion of positively stained tumor cells in 5 high power fields (scored as 0, <5%; 1, 5–25%; 2, 26–50%; 3, 51–75%; 4, >75%). The sum of the staining intensity score and score on the percentage of positive tumor cells was graded as follows: −, 0–1; 1+, 2–3; 2+, 4–5; and 3+, 6–7. The results of IHC were judged independently by two investigators (Y.I. and R.N.) who were unaware of the clinical data, and consensus was reached for any discordant cases.

### Statistical analyses

Correlations between protein expression and characteristics were assessed by the Fisher’s exact test. Overall survival (OS) was calculated from the date of surgery. Kaplan-Meier survival curves were drawn for OS and compared by log-rank test. Univariate and multivariate analyses were performed using the COX regression model. Statistical significance was set at P<0.05 for each analysis. All statistical analyses were carried using the IBM SPSS Statistics version 21 (IBM SPSS, Inc., Armonk, NY, USA).

## Results

### TMA analysis of Chinese patients

**Figure 1:**
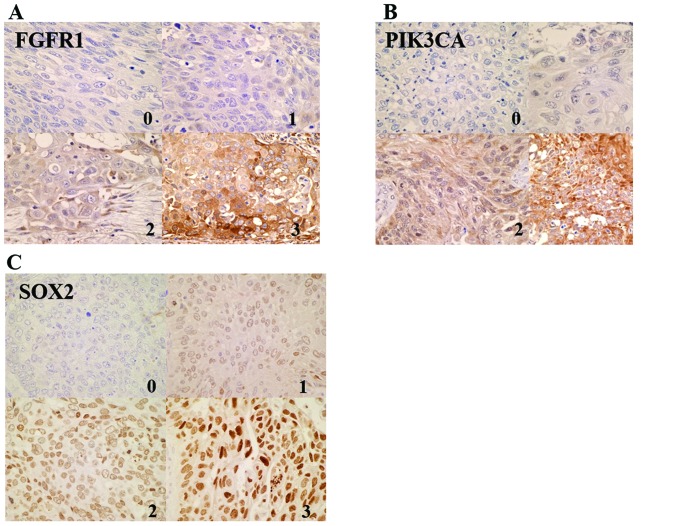
Immunohistochemical staining for FGFR1, PIK3CA and SOX2. (A–C) Immunohistochemical staining for FGFR1, PIK3CA and SOX2 protein expression in tumor cells. Scores 0, 1, 2 and 3 correspond to negative, weak, moderate and strong staining, respectively (objective magnification, ×40).

Fifty-seven Chinese SQCC specimens were available for IHC analysis. Six specimens (11%) were observed to be FGFR1-positive (Table I, Fig. 1A). Forty-five (82%) of 57 SQCC patients were positive for PIK3CA (Table I, Fig. 1B). High SOX2 expression was observed in 40 (70%) of 57 SQCC specimens (Table I, Fig. 1C). The results of correlation analysis between FGFR1, PIK3CA and SOX2 protein expression and patients’ characteristics are summarized in Table I. FGFR1 expression status was found to be related to gender and T factors.

### Association between FGFR1, PIK3CA and SOX2 expression and prognosis in Chinese SQCC specimens

**Figure 2:**
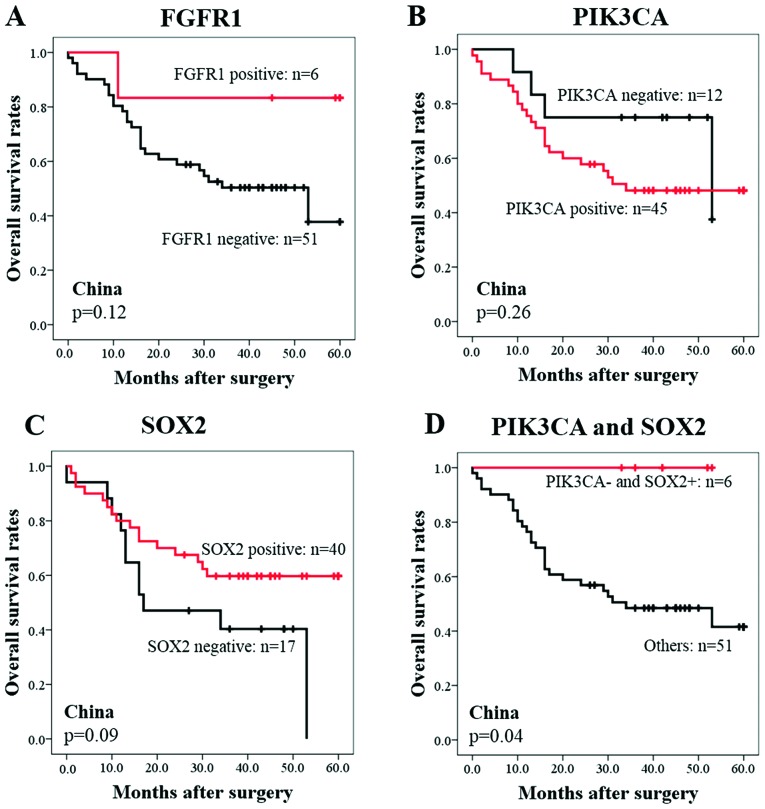
Kaplan-Meier analyses of overall survival in the Chinese training cohort. The significance of the difference in overall survival between subgroups was analyzed by log-rank test. (A) Overall survival curves of patients with positive (red) or negative (black) expression of FGFR1. (B) Overall survival curves of patients with positive (red) or negative (black) expression of PIK3CA. (C) Overall survival curves of patients with positive (red) or negative (black) expression of PIK3CA. (D) Overall survival curves of patients with PIK3CA-negative and SOX2-positive staining (PIK3CA^−^/SOX2^+^) (red) or others (black). The overall survival was significantly longer in the PIK3CA^−^/SOX2^+^ group than in the others (P<0.04).

We next evaluated the prognostic significance of the expression of three proteins by IHC. FGFR1-positive or PIK3CA-negative or SOX2-positive status showed trends toward better survival, although the differences were not statistically significant (P=0.12, 0.26, and 0.09, respectively) (Fig. 2A–C). Therefore, we next used a combination of expression of two proteins by IHC to improve the prognostic classification of SQCC patients. Among all combinations, the combination of PIK3CA and SOX2 was the best classification distinguishing good prognosis from poor prognosis. Patients with PIK3CA-negative and SOX2-positive staining (PIK3CA^−^/SOX2^+^) showed good prognosis compared to those with PIK3CA-positive or SOX2-negative staining (Fig. 2D). The 5-year survival rate among the cases was 100% (Fig. 2D). In contrast, those with PIK3CA-positive or SOX2-negative staining had significantly worse survival than the low-risk PIK3CA^−^/SOX2^+^ group (P=0.04) (Fig. 2D). The 5-year survival rate was 42% among the cases in the PIK3CA-positive or SOX2-negative group (Fig. 2D).

### Validation of the prognostic significance of SOX2 and PIK3CA

We next examined the robustness of the PIK3CA^−^/SOX2^+^ immunohistological profile for classifying patients into prognostic groups in two independent sets of specimens from American TMA and 66 Japanese SQCC patients who had undergone surgical resection. Unfortunately, the prognostic significance of PIK3CA^−^/SOX2^+^ could not be validated in the USA cohort, because only 1 of the 52 USA cases was PIK3CA^−^/ SOX2^+^. In the Japanese cohort, negative immunoreactions of PIK3CA were found in 56 (85%) of 66 cases and positive immunoreactions of SOX2 were observed in 45 (68%) of 66 cases (Table II). The associations between PIK3CA and SOX2 expression levels and the patient characteristics are shown in Table II. SOX2 expression status was related to smoking status (P=0.01).

**Figure 3:**
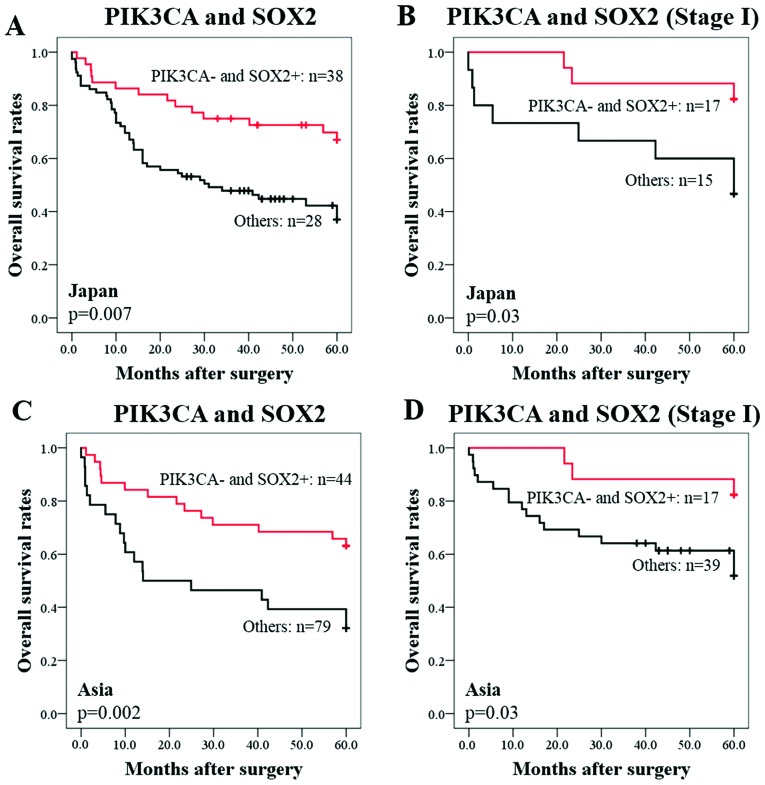
Kaplan-Meier analyses of overall survival in Japanese SQCC patients as a validation set. The significance of the difference in overall survival between subgroups was analyzed by log-rank test. (A) Overall survival curves of Japanese patients with PIK3CA^−^/SOX2^+^ (red) or others (black). The overall survival was significantly longer in patients with PIK3CA^−^/SOX2^+^ than in the others (P=0.007). (B) Overall survival curves of Japanese stage I patients with PIK3CA^−^/SOX2^+^ (red) or other Japanese stage I patients (black). The overall survival was significantly longer in patients with PIK3CA^−^/SOX2^+^ than in the others among Japanese patients with stage I (P=0.03). (C) Overall survival curves of Asian patients with PIK3CA^−^/SOX2^+^ (red) or others (black). The overall survival was significantly longer in Asian patients with PIK3CA^−^/SOX2^+^ than in the others among Asian patients (P=0.002). (D) Overall survival curves of Asian stage I patients with PIK3CA^−^/SOX2^+^ (red) or others (black). The overall survival was significantly longer in the PIK3CA^−^/SOX2^+^ group than in the others among Asian stage I patients (P=0.03).

The 5-year survival rates were 54% among patients with PIK3CA-negative and 30% among patients with PIK3CA-positive status (data not shown). The 5-year survival rates were 56% among patients with SOX2-positive and 38% among patients with SOX2-negative status (data not shown). However, these differences were not statistically significant (P=0.13 and P=0.12, respectively). Kaplan-Meier survival analysis showed that the 38 PIK3CA^−^/SOX2^+^ cases had significantly favorable survival than the other 28 cases (P=0.007) (Fig. 3A). The 5-year survival rates were 63% among patients with PIK3CA^−^/SOX2^+^ and 32% among the other patients (Fig. 3A). Furthermore, among the 32 patients with stage I SQCC, the PIK3CA^−^/SOX2^+^ cases (n=17) had significantly better survival than the group with PIK3CA-positive or SOX2-negative status (n=15) (P=0.03) (Fig. 3B). The 5-year survival rates were 82% among the PIK3CA^−^/SOX2^+^ cases and 47% among the group with PIK3CA positive or SOX2-negative status (Fig. 3B). Among the 123 Asian (Chinese and Japanese) patients with SQCC, the group with PIK3CA-positive or SOX2-negative status (n=79) had significantly poorer survival than the PIK3CA^−^/SOX2^+^ cases (n=44) (P=0.002) (Fig. 3C). The 5-year survival rates were 70% among the PIK3CA^−^/SOX2^+^ cases and 40% among the group with PIK3CA-positive or SOX2-negative status (Fig. 3C). Furthermore, among 56 Asian stage I patients, the 5-year survival rates were 82% among the PIK3CA^−^/SOX2^+^ cases and 52% among the group with PIK3CA-positive or SOX2-negative status (P=0.03) (Fig. 3D). Notably, this classification correctly predicted poor survival in 16 (89%) of the 18 stage I cases who had died during the 5-year follow-up period (Fig. 3D).

### Univariate analysis and multivariate analysis

Finally, we investigated whether the prognostic ability of PIK3CA^+^/SOX2^−^ expression was affected by underlying clinical covariates by performing univariate and multivariable Cox proportional hazards survival analyses. Univariate analysis of the 123 Asian patients revealed that age, T factor, N factor, p-stage and PIK3CA^−^/SOX2^+^ classification (HR for death in the high-risk PIK3CA-positive or SOX2-negative group compared with the low-risk reference PIK3CA^−^/SOX2^+^ group = 2.47; 95% CI, 1.35–4.51; P=0.003) were statistically significant predictors of survival (Table III). Multivariable analysis of the 123 patients, adjusted for age, T factor, N-factor, p-stage and PIK3CA/SOX2 classification, showed that age, N factor and PIK3CA/SOX2 classification were statistically significant (HR, 1.99, 2.98 and 2.50; 95% CI; 1.05–3.77, 1.23–7.22 and 1.35–4.65; P=0.04, 0.02 and 0.004, respectively). We also performed univariate and multivariable survival analyses of the 56 Asian patients with stage I. Univariate analysis revealed that PIK3CA/SOX2 classification (HR for death in the PIK3CA-positive or SOX2-negative group compared with the low-risk reference group = 3.51; 95% CI, 1.02–12.08; P<0.05) was significantly associated with survival (Table III). In the multivariable Cox proportional hazards model of Asian stage I patients, the PIK3CA/SOX2 classification was significantly associated with survival and was an independent prognostic factor among the stage I cases (HR for death in the high-risk PIK3CA/SOX2 expression group compared with the low-risk reference group = 4.23; 95% CI, 1.17–15.4; P=0.03). The combination of PIK3CA and SOX2 expression identified Asian stage I lung SQCC patients who have a poor prognosis.

## Discussion

In the present study, we examined the prognostic significance of FGFR1, PIK3CA and SOX2 protein expression by IHC in SQCC. We ultimately developed a prognostic model which was based on the combination status of PIK3CA/SOX2 expression to predict the prognosis of Asian patients with stage I SQCC. PIK3CA and SOX2 genes are located on chromosome 3q26. Recent whole genomic studies indicated frequent amplification at chromosome 3q26 in SQCC, suggesting a potential critical role of this chromosome in carcinogenesis and progression in SQCC (20,21).

PIK3CA regulates the phosphatidylinositol 3-kinase (PI3K)/Akt signaling pathway which is critical for cell survival of human cancer (25–27). PIK3CA gene alteration was found in 16% of lung SQCC samples by The Cancer Genome Atlas, resulting in PI3K pathway alterations (17). In addition, PIK3CA gene alteration was more frequently observed in lung SQCC than in ADC (28,29). In Asian patients, PIK3CA copy number gains by FISH were found in 43% of Japanese SQCC patients (29). PIK3CA amplification was also observed in 42% of Chinese SQCC patients (30). These results are consistent with comparative genomic hybridization studies of SQCC showing consistent gains in chromosome 3q26 including the PIK3CA gene. The association between PIK3CA gene alteration and the patient prognosis has not been clarified; however, patients with PIK3CA gene mutation tended to have an unfavorable prognosis (29). In the present study, patients with PIK3CA-negative status showed a trend toward better survival compared with patients with PIK3CA-positive staining. These findings suggest that PIK3CA alteration may be involved in the process of carcinogenesis of SQCC and contribute to the prognosis of SQCC.

SOX2 is a transcription factor essential for early mammalian development and maintenance of stem cells in multiple adult tissues (31). SOX2 is also important for maintenance of lung epithelium cells, Clara cells, ciliated cells, and goblet cells which have known roles in squamous cell differentiation (32). SOX2 and TP63 play roles in squamous cell differentiation. SOX2-positive and p63-negative immunohistochemical staining correlated with high-grade histology across NSCLC subtypes (33). Recently, SOX2 has been shown as an oncogene activated by 3q26.3 amplification in lung SQCC (20,34). The Cancer Genome Atlas Research reported that overexpression and amplification of SOX2 were observed in 21% of SQCC samples (17). SOX2 was also highly overexpressed in lung SQCC when compared with ADC by IHC (31). As to prognostic significance, SOX2 amplification or overexpression showed a trend toward better survival (31,35). SOX2-positive status showed a trend toward better prognosis in this study, which is consistent with findings from previous studies. The association between high SOX2 expression and favorable prognosis is unclear; however, SOX2 might be implicated in the control of cell differentiation toward the formation of squamous metaplasia and well-differentiated SQCC (36,37). Low SOX2 expression tended to be associated with poorly-differentiated SQCC tumors (38). In our analysis using the Japanese cohort, 9 of the 10 Grade 1 SQCC cases showed SOX2-positive expression. Therefore, the role of SOX2 in squamous cell differentiation might affect the prognosis of SQCC patients.

A limitation of this study is validation of the American cohort. The prognostic significance of PIK3CA^−^/SOX2^+^ could not be confirmed in the American cohort, because there was only one patient with PIK3CA^−^/SOX2^+^. The frequency of driver mutations seems to be different in Asian and Caucasian lung AC patients. EGFR mutations are found at different frequencies in Caucasian and East Asian patients with lung AC (39). Although ethnic differences in the frequency of driver mutations in lung SQCC were not clarified, the differences seem to contribute to the incidence and prognosis of lung SQCC.

In conclusion, we found that SQCCs with PIK3CA^−^/SOX2^+^ showed a trend towards better survival in Japanese and Chinese SQCC patient cohorts. Notably, PIK3CA/SOX2 classification, which was based on IHC expression, was useful for identifying stage I SQCC patients who are at high risk of death. Patients in the high-risk stage I group classified by PIK3CA/SOX2 expression pattern may be candidates for adjuvant therapy in SQCC. Therefore, patient selection based on PIK3CA/SOX2 expression offers the potential to improve survival in SQCC patients. The significance of PIK3CA/ SOX2 classification will be further validated in a large-scale SQCC cohort.

## Figures and Tables

**Table I:** Immunohistochemical analysis of Chinese SQCC specimens.

			FGFR1 expression					PIK3CA expression					SOX2 expression				
								
			Positive		Negative			Positive		Negative			Positive		Negative		
											
Variables	N	%	N	%	N	%	P-value	Nl	%	N	%	P-value	N	%	N	%	P-value
Total	57		6		51			45		12			40		17		
Gender																	
Male	53	93	3	50	50	98		41	91	12	100		37	93	16	94	
Female	4	7	3	50	1	2	0.003	4	9	0	0	1	3	8	1	6	1.00
Age (years)																	
<65	23	40	4	67	19	37		18	40	5	42		16	40	7	41	
≥65	34	60	2	33	32	63	0.21	27	60	7	58	1	24	60	10	59	1.00
Stage																	
I	24	42	4	67	20	39		19	42	5	42		16	40	8	47	
II+III	33	58	2	33	31	61	0.23	26	58	7	58	1	24	60	9	53	0.77
Grade																	
G1	7	12	2	33	5	10		7	16	0	0		4	10	3	18	
G2+G3	50	88	4	67	46	90	0.15	38	84	12	100	0	36	90	14	82	0.42
T factor																	
T1	5	9	3	50	2	4		5	11	0	0		5	13	0	0	
T2+T3	52	91	3	50	49	96	0.006	40	89	12	100	1	35	88	17	100	0.31
N factor																	
N0	34	60	5	83	29	57		26	58	8	67		25	63	9	53	
N1+N2	23	40	1	17	22	43	0.39	19	42	4	33	1	15	38	8	47	0.56

**Table II:** Associations between PIK3CA and SOX2 expression levels and the patient characteristics.

			PIK3CA expression					SOX2 expression				
						
			Positive		Negative			Positive		Negative		
								
Variables	N	%	N	%	N	%	P-value	N	%	N	%	P-value
Total	66		10		56			45		21		
Age (years)												
<65	13	20	2	20	11	20		10	22	3	14	
≥65	53	80	8	80	45	80	0.71	35	78	18	86	1.00
Gender												
Male	58	88	9	90	49	88		42	93	16	76	
Female	8	12	1	10	7	13	1.00	3	7	5	24	0.10
Smoking status												
Ever-smoker	60	91	9	90	51	91		44	98	16	76	
Never smoker	6	9	1	10	5	9	1.00	1	2	5	24	0.01
T factor												
T1	17	26	3	30	14	25		14	31	3	14	
T2–4	49	74	7	70	42	75	0.71	31	69	18	86	0.23
N factor												
N0	41	62	7	70	34	61		27	60	14	67	
N1–2	25	38	3	30	22	39	0.73	18	40	7	33	0.79
Stage												
I	32	48	6	60	26	46		21	47	11	52	
II–III	36	55	4	40	30	54	0.51	24	53	10	48	0.79
Grade												
G1	10	15	2	20	8	14		9	20	1	5	
G2–3	56	85	8	80	48	86	0.64	36	80	20	95	0.15
ly factor												
ly0	17	26	3	30	14	25		15	33	2	10	
Ly1–3	49	74	7	70	42	75	0.71	30	67	19	90	0.07
v factor												
v0	26	39	3	30	23	41		20	44	6	29	
v1–3	40	61	7	70	33	59	0.73	25	56	15	71	0.28
Adjuvant chemotherapy (UFT)												
Yes	28	42	3	30	25	45	22	49	6	29		
No	38	58	7	70	31	55	0.31	23	51	15	71	0.10

**Table III:** Univariate and multivariable Cox proportional hazards models of factors associated with death for all Asian patients and for the stage I patients.

		Univariate analysis			Multivariate analysis		
			
Characteristics	Comparison	HR	95% CI	P-value	HR	95% CI	P-value
All cases (n=123)							
Age (years)	<65 vs. ≥65	1.93	1.02–3.62	0.04	1.99	1.05–3.77	0.04
Gender	Male vs. female	1.24	0.50–3.11	0.65			
T factor	T1 vs. T2–4	2.65	1.13–6.20	0.03			0.37
N factor	N0 vs. N1–2	3.01	1.79–5.07	<0.001	2.98	1.23–7.22	0.02
p-Stage	I vs. II–III	2.19	1.28–3.76	0.005			0.88
Grade	G1 vs. G2+G3	0.76	0.39–1.50	0.43			
SOX2/PIK3CA expression	SOX2^+^/PIK3CA^−^ vs. others	2.47	1.35–4.51	0.003	2.50	1.35–4.65	0.005
Stage I cases (n=56)							
Age (years)	<65 vs. ≥65	2.09	0.70–6.25	0.18			0.08
Gender	Male vs. female	1.35	0.31–5.83	0.69			0.68
T factor T1 vs.	T2–4	1.50	0.57–3.94	0.41			0.94
Grade G1 vs.	G2+G3	1.67	0.39–7.20	0.49			0.57
SOX2/PIK3CA expression	SOX2^+^/PIK3CA^−^ vs. others	3.51	1.02–12.08	0.05	4.23	1.17–15.37	0.03

HR, hazard ratio; CI, confidence interval.
